# Prophylactic administration of lecithinized superoxide dismutase for a murine model of oxaliplatin-induced myelosuppression

**DOI:** 10.3389/fphar.2025.1607814

**Published:** 2025-07-22

**Authors:** Mikako Shimoda, Akari Yamaguchi, Ayano Shikata, Yusuke Murakami, Masahiro Kawahara, Tohru Mizushima, Ken-ichiro Tanaka

**Affiliations:** ^1^ Laboratory of Bio-Analytical Chemistry, Research Institute of Pharmaceutical Sciences, Faculty of Pharmacy, Musashino University, Nishi-Tokyo, Japan; ^2^ LTT Bio-Pharma Co., Ltd, Tokyo, Japan; ^3^ Laboratory of Drug Repositioning, Faculty of Pharmaceutical Sciences, Sojo University, Kumamoto, Japan

**Keywords:** lecithinized superoxide dismutase, leukopenia, myelosuppression, oxaliplatin, mice, reactive oxygen species

## Abstract

**Background:**

Oxaliplatin, in combination with 5-fluorouracil and leucovorin, is a standard treatment for colorectal cancer and shows high efficacy. However, oxaliplatin induces side effects, such as chemotherapy-induced peripheral neuropathy and myelosuppression, which may lead to dose reduction, temporary drug withdrawal, or discontinuation. Lecithinized superoxide dismutase (PC-SOD) is a drug delivery system formulation with improved blood stability and tissue affinity for SOD. A phase II clinical trial of PC-SOD for chemotherapy-induced peripheral neuropathy has been conducted, and its efficacy has been confirmed for certain parameters.

**Methods:**

In this study, we focused on myelosuppression, a major side effect of oxaliplatin, and aimed to elucidate the preventive effect of PC-SOD in a murine model of myelosuppression.

**Results:**

Oxaliplatin administration decreased the white blood cell, platelet, and red blood cell counts and hemoglobin levels in the whole blood of mice. PC-SOD treatment significantly restored the oxaliplatin-dependent reduction in white blood cell count (day 10). The gene expression of cytokines involved in hematopoietic progenitor cell differentiation and proliferation, including colony-stimulating factor (CSF)2, CSF3, interleukin (IL)-3, IL-4, IL-5, IL-6, IL-9, and stem cell factor, was also decreased by oxaliplatin administration. In contrast, PC-SOD treatment markedly restored the gene expression of these cytokines. *In vivo* imaging analysis showed that oxaliplatin treatment enhanced reactive oxygen species (ROS) production in the femur and tibia, whereas PC-SOD significantly suppressed this production. Furthermore, analysis of mouse-derived bone marrow cells revealed that PC-SOD suppressed oxaliplatin-induced cytotoxicity and ROS production *in vitro*.

**Conclusion:**

These results suggest that PC-SOD exerts an antioxidant effect and prevents oxaliplatin-induced myelosuppression, particularly in a murine model of leukopenia.

## Graphical Abstract

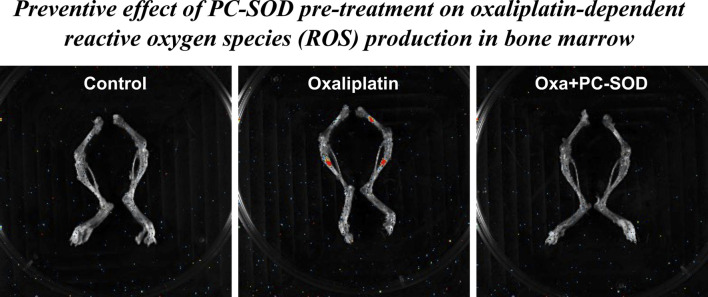

## Introduction

Oxaliplatin is a platinum-based drug that inhibits DNA replication and transcription by binding to DNA strands in cancer cells and forming platinum-DNA crosslinks. Oxaliplatin is used to treat gastric and small intestinal cancers, as well as unresectable colon, rectal, and pancreatic cancers (Rottenberg et al., 2021). The FOLFOX protocol, which combines oxaliplatin with 5-fluorouracil and leucovorin, is the standard treatment for these cancers (Comella et al., 2009). However, oxaliplatin causes peripheral neuropathy with symptoms like numbness and pain in limbs and mouth, and myelosuppression, including thrombocytopenia, leukopenia, and neutropenia, as major adverse effects (Burz et al., 2009; Yu et al., 2021; Mattar et al., 2024). These effects can lead to reduced oxaliplatin dosage or treatment suspension. Thus, compounds that prevent these effects while maintaining the anticancer activity of oxaliplatin may aid in the development of new treatment protocols.

Studies have shown that oxaliplatin treatment increases oxidative stress, including reactive oxygen species (ROS) overproduction. In mouse experiments, increased intracellular ROS production and levels of thiobarbituric acid reactive substances and 4-hydroxynonenal, indicators of lipid peroxidation, were observed in spinal cord tissue after 15 days of oxaliplatin treatment (Agnes et al., 2023). After 28 days of oxaliplatin administration to rats, malondialdehyde (MDA), a marker of lipid peroxidation, increased in the spinal cord, while antioxidant enzymes, including superoxide dismutase (SOD), glutathione peroxidase, and catalase, decreased, indicating increased oxidative stress (Zhang et al., 2020). Intraperitoneal oxaliplatin administration in mice increased MDA levels in the liver and decreased SOD, catalase, and glutathione levels, suggesting that oxidative stress may contribute to hepatotoxicity (Cheng et al., 2024). While no studies have measured oxidative stress in animal models of oxaliplatin-induced myelosuppression, findings have been reported for cisplatin, another platinum-based anticancer drug. Cisplatin treatment increases intracellular ROS production and levels of 8-hydroxydeoxyguanosine, a marker of oxidative DNA damage, in mouse bone marrow cells (Xu et al., 2023). Thus, we speculated that increased oxidative stress is involved in oxaliplatin-induced adverse effects, including myelosuppression.

SOD breaks down superoxide anions into oxygen and hydrogen peroxide, which are detoxified by catalase and glutathione peroxidase, respectively. SOD is essential for antioxidant defense in organisms exposed to oxidative stress (Carillon et al., 2013; Younus, 2018). SOD breaks down superoxide anions into oxygen and hydrogen peroxide, which are detoxified by catalase and glutathione peroxidase (McCord and Fridovich, 1988; Carillon et al., 2013; Younus, 2018). Although SOD is a therapeutic target for oxidative stress disorders, its clinical use remains limited because of its low stability in the body. To address this issue, we engineered lecithinized SOD (PC-SOD) by incorporating four phosphatidylcholine derivatives bound to each SOD dimer (Igarashi et al., 1992). PC-SOD shows improved plasma stability and tissue affinity compared to unmodified SOD (Igarashi et al., 1994; Broeyer et al., 2008; Tanaka et al., 2010). We have demonstrated the efficacy of PC-SOD in various disease models, including chronic obstructive pulmonary disease, idiopathic pulmonary fibrosis, ulcerative colitis, renal ischemia/reperfusion injury, and acute respiratory distress syndrome (Ishihara et al., 2009; Tanaka et al., 2010; Tanaka et al., 2011; Tanaka et al., 2012a; Tanaka et al., 2012b; Tanaka et al., 2017; Tanaka et al., 2022b). PC-SOD is expected to prevent diseases and effects associated with excessive production of ROS.

A recent study showed that prophylactic PC-SOD administration was effective against oxaliplatin-dependent peripheral neuropathy in rats (Qiao et al., 2024). Therefore, we aimed to elucidate the prophylactic effects of intravenous PC-SOD on oxaliplatin-induced myelosuppression, particularly leukopenia, using a murine model. We analyzed the preventive effect of PC-SOD, focusing on cytokine expression in hematopoietic progenitor cell differentiation and proliferation and ROS production in mouse bone marrow. The preventive effects of PC-SOD were analyzed using bone marrow cells from ICR mice.

## Materials and methods

### Chemicals

PC-SOD (3,000 U/mg) was obtained from the laboratory stock (Igarashi et al., 1992). Oxaliplatin (Code: 4291410A2149) was obtained from NIPRO CORPORATION (Tokyo, Japan). The luminol-based chemiluminescent probe L-012 (Code: 120–04891), RPMI 1640 medium (Code: 189–02025), and isoflurane (Code: 099–06571) were obtained from Fujifilm Wako Pure Chemical Corporation (Tokyo, Japan). Hydrogen peroxide chemiluminescent detection kit (Code: ADI-907-012) was purchased from Enzo Life Sciences (Farmingdale, NY, United States). Fetal bovine serum (Code: 10270106) was purchased from Thermo Fisher Scientific, and EDTA-2K (Code: 340–01511) was purchased from Dojindo Laboratories (Kumamoto, Japan). CellTiter-Glo^®^ 2.0 (Code: G9242) was purchased from Promega Corporation (Madison, WI, United States), and 2′,7′-dichlorodihydrofluorescein diacetate (H_2_DCFDA) (Code: D6883) was obtained from Merck KGaA (Darmstadt, Germany). ISOGEN (Code: 311–02501) was obtained from Nippon Gene (Toyama, Japan), ReverTra Ace^®^ qPCR RT Master Mix (Code: FSQ-201) was purchased from Toyobo (Osaka, Japan), and KAPA SYBR Fast qPCR Kits (Code: KK4602) were obtained from Nippon Genetics (Tokyo, Japan).

### Animals

Male ICR mice (6–7 weeks old) were purchased from Charles River (Yokohama, Japan) and kept in dedicated cages (maximum 4 mice/cage) in individual ventilation systems (MVCS-140, ITEC Co., Ltd., Tokyo, Japan) under a 12 h light/dark cycle and fed a controlled diet (MF, Oriental Yeast Co., Ltd., Tokyo, Japan). The experiments and procedures were performed in accordance with the Guide for the Care and Use of Laboratory Animals, as adopted and promulgated by the National Institutes of Health (Bethesda, MD, United States), and were approved by the Animal Care Committee of Musashino University (approval number 09-A-2024). The animal studies in this manuscript were based on humane endpoints.

### Administration of oxaliplatin or PC-SOD and measurement of hematological parameters

Male ICR mice (8–9 weeks old) weighing approximately 35 g were used for the animal experiments. PC-SOD (3000 U/kg = 1.0 mg/kg) was dissolved in 0.45% sterile NaCl solution and administered intravenously once daily from 1 day before oxaliplatin administration (day 0) to day 4. Oxaliplatin (12.5 mg/kg) was dissolved in sterile saline and administered only once intraperitoneally 5 min after day 1 PC-SOD administration. The dose of oxaliplatin that induced leukopenia was determined from previous studies (Karlsson et al., 2012). In addition, the dose of PC-SOD was determined based on previous studies, specifically the dose at which efficacy analyses were conducted for oxaliplatin-induced peripheral neuropathy (Tanaka et al., 2017; Tanaka et al., 2022b; Qiao et al., 2024).

**SCHEME 1:**
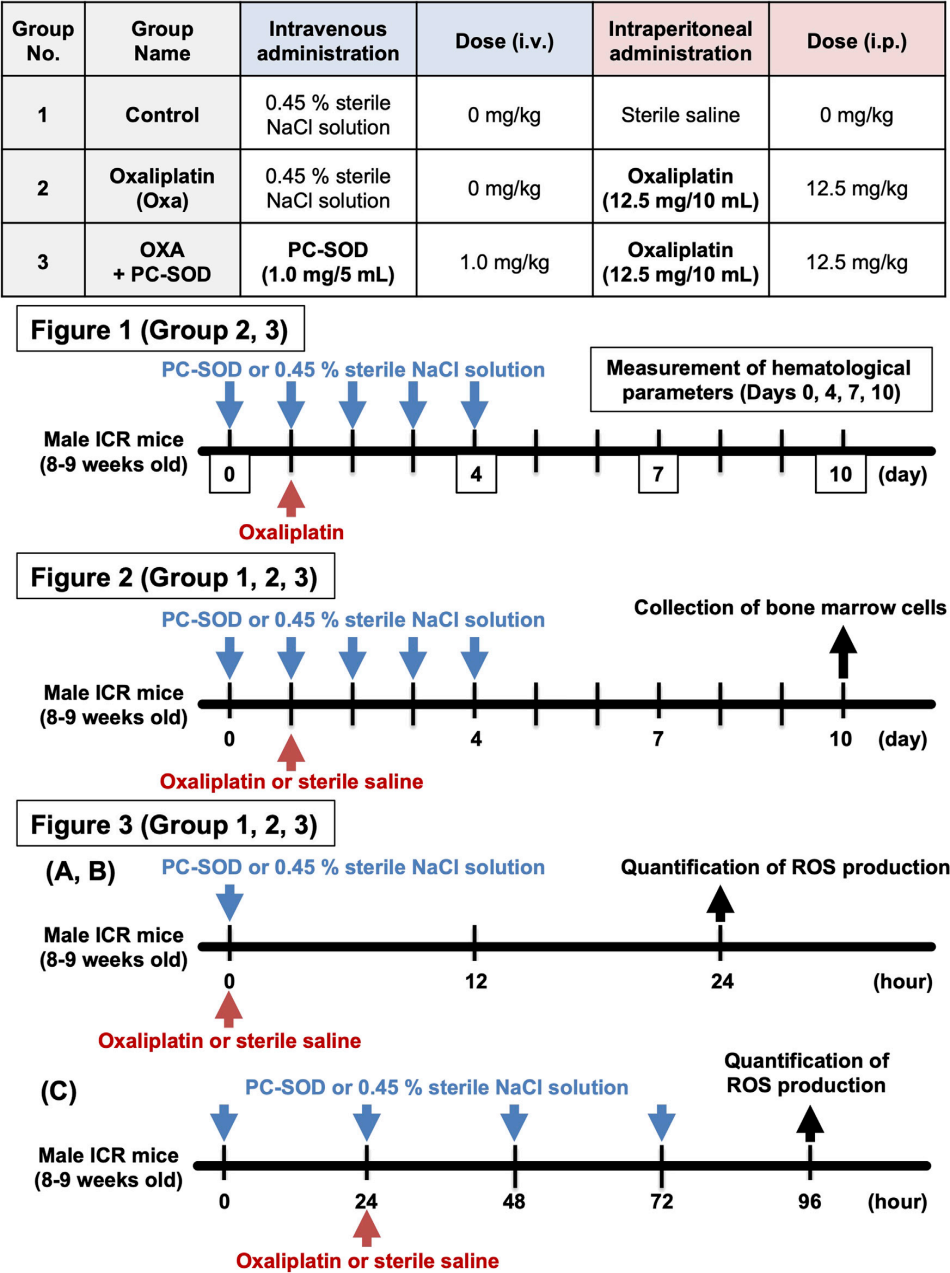
An overview of the animal experiments in Figures 1–3.

On days 0, 4, 7, and 10, mice were anesthetized using a mixture of 0.75 mg/kg medetomidine, 4.0 mg/kg midazolam, and 5.0 mg/kg butorphanol on days 0, 4, 7, and 10. After anesthesia, whole blood was collected from the tail vein using an EDTA-coated blood collection tube (Code: 2909000; Paul Marienfeld, Lauda-Königshofen, Germany). An automated hematology analyzer (MEK-6450, Nihon Kohden, Tokyo, Japan) was used to measure the white blood cell (WBC), platelet (PLT), and red blood cell (RBC) counts and hemoglobin (HGB) levels in whole blood. Please refer to Scheme 1 for the protocol for grouping and time course of animal experiments.

### Real-time RT-PCR

Mice were euthanized, and the bones of both legs were isolated 9 days after oxaliplatin administration (day 10). Total RNA was extracted from bone marrow cells using ISOGEN, following the manufacturer’s protocol. Pure RNA was purified from crude RNA, and cDNA was synthesized using the reverse transcriptase, ReverTra Ace^®^ qPCR RT Master Mix. Real-time RT-PCR was performed using the KAPA SYBR Fast qPCR Kit and analyzed using the CFX96™ Real-Time System (Bio-Rad, Hercules, CA, United States) and CFX Manager™ software (version 3.1, Bio-Rad). Primers were designed using the Primer-BLAST website (https://www.ncbi.nlm.nih.gov/tools/primer-blast/). The primer sequences are described in Supplementary Figure S1. Glyceraldehyde-3-phosphate dehydrogenase (GAPDH) mRNA was used as an internal standard. Gene expression data in each figure are shown relative to the control value of 1.00.

### Quantification of ROS production

ROS production in the bone marrow of mice was measured using the FUSION chemiluminescence imaging system (Vilber Lourmat, Collégien, France), as previously described (Tanaka et al., 2017; Tanaka et al., 2021; Tanaka et al., 2022a; Tanaka et al., 2022b). At 24 h after intraperitoneal administration of oxaliplatin or sterile saline, mice were subcutaneously administered L-012 (75 mg/kg), a ROS-sensing chemiluminescence probe, dissolved in sterile saline. After 20 min, the bones of both legs were removed from the mice, and the muscle and fatty tissues were carefully stripped from the bones. The cleaned bones were placed in plastic Petri dishes, and brightfield and chemiluminescence images (5-min exposure) were obtained using a FUSION chemiluminescence imaging system. The superimposition of bright-field and chemiluminescence images and quantification of chemiluminescence were performed using the FUSION chemiluminescence imaging system software (version 18.02, Vilber Lourmat).

### Collection and analysis of bone marrow cells from mice

Bone marrow cells were harvested from the bones of both legs of untreated healthy mice. After isolating the bones of both legs for ROS measurement, RPMI1640 medium containing 10% heat-inactivated FBS was injected into the medullary cavity of each bone, and the bone marrow cells were washed out. A cell strainer with a mesh size of 40 µm (Code: VCS-40, AS ONE Corporation, Osaka, Japan) was used to remove tissue fragments and other impurities. RBCs were lysed using RBC Lysis Buffer (Code: 420301, BioLegend, San Diego, CA, United States), and bone marrow cells were seeded in RPMI1640 medium containing 10% heat-inactivated FBS and 50 μmol/L 2-mercaptoethanol (Code: 21438–82, Nacalai Tesque, Kyoto, Japan) in 96-well plates.

The cells were pre-treated with PC-SOD, followed by oxaliplatin treatment. After 24 h, cell viability was measured using CellTiter-Glo^®^ 2.0, which emits chemiluminescence in reaction with intracellular ATP. Intracellular ROS levels were measured using H_2_DCFDA (10 μmol/L). Measurements were obtained using a microplate reader (Tecan, Kawasaki, Japan).

### Statistical analysis

**FIGURE 1:**
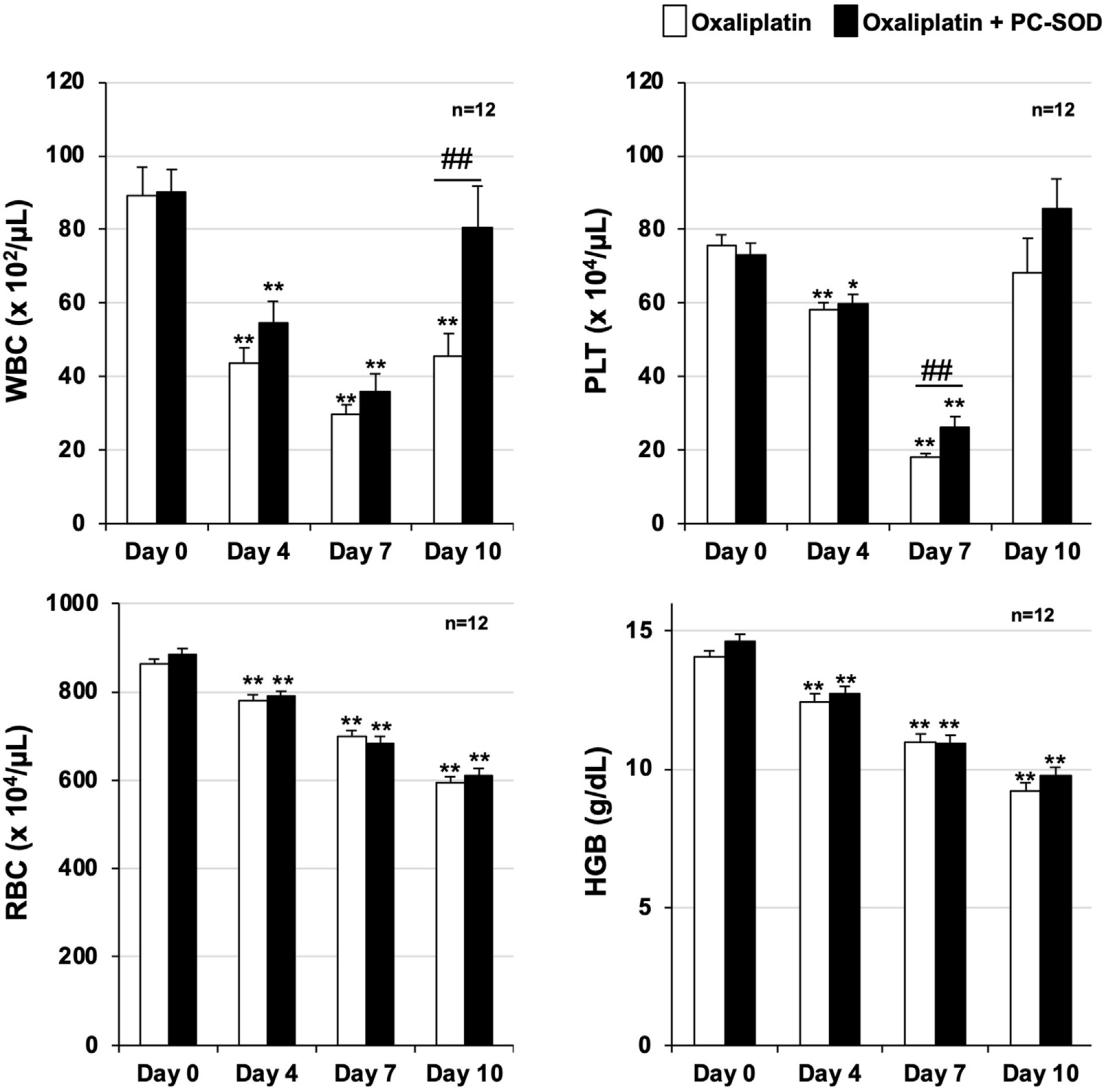
Preventive effect of PC-SOD pre-treatment on oxaliplatin-induced leukopenia PC-SOD (3 kU/kg) or 0.45% NaCl was administered intravenously to ICR mice once daily, five times during days 0–4. Oxaliplatin (12.5 mg/kg) was intraperitoneally administered to the mice on day 1. The hematological parameters of ICR mice were analyzed using an automated hematology analyzer, Celltac α (MEK-6450). White blood cell (WBC), platelet (PLT), and red blood cell (RBC) counts, and hemoglobin (HGB) levels are shown. Data are shown as mean ± S.E.M. (n = 12); #*P* < 0.05; ** or ##*P* < 0.01 [* vs. Day 0 (Steel’s multiple comparison test); # Oxa vs. PC (Mann–Whitney U test)].

**FIGURE 2:**
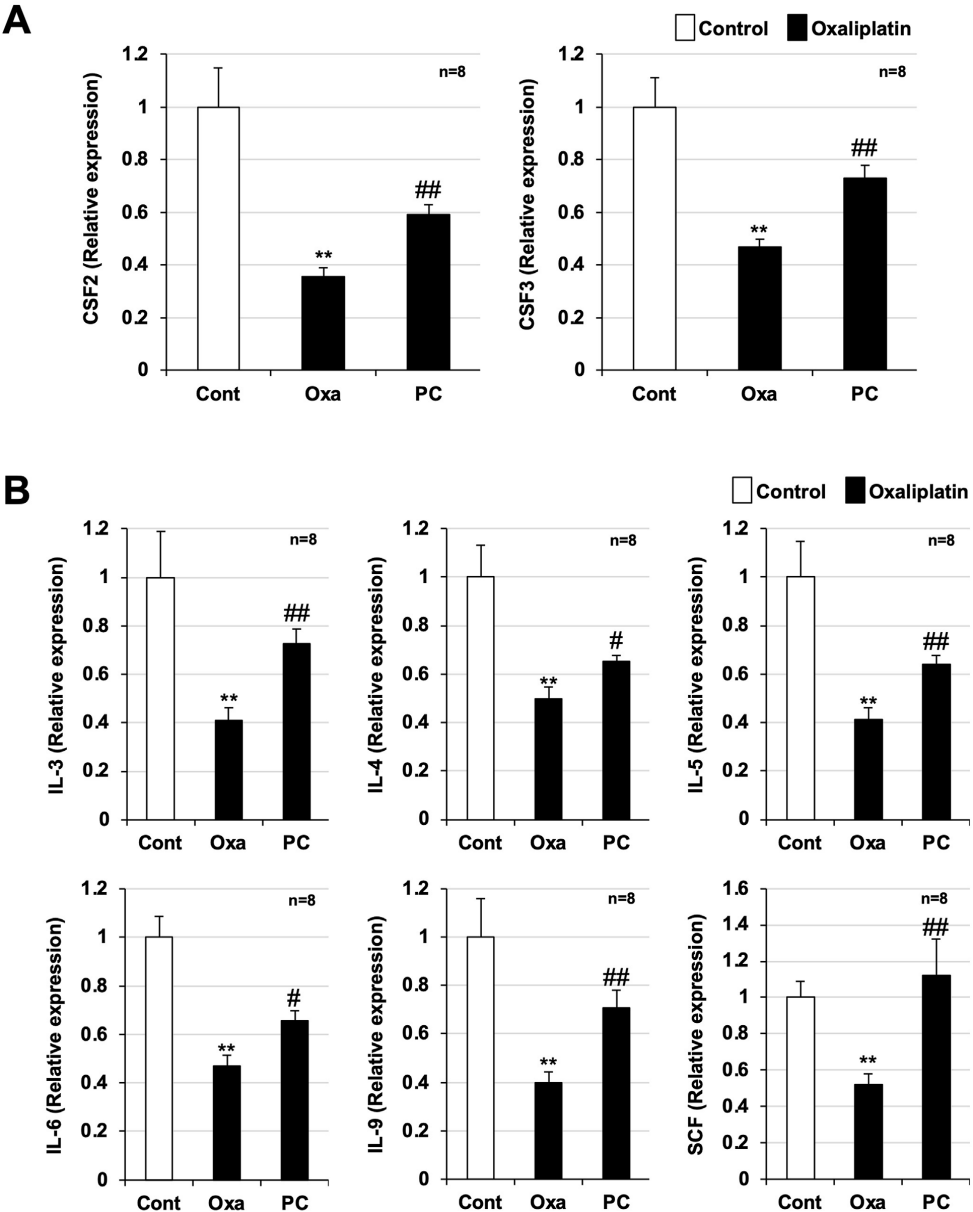
Preventive effect of PC-SOD pre-treatment on the reduction of cytokines involved in bone marrow cell proliferation and differentiation. PC-SOD (PC, 3 kU/kg) or 0.45% NaCl was administered intravenously to ICR mice once daily, five times during days 0–4. Oxaliplatin (Oxa, 12.5 mg/kg) or sterile saline (Cont) was administered intraperitoneally to mice once on day 1. Bone marrow cells were harvested from the femurs of the mice 9 days after oxaliplatin administration (Day 10). RNA was extracted and purified from the cells, and cDNA was synthesized. cDNA was subjected to real-time RT-PCR with a specific primer set. **(A)**: CSF2 and CSF3, **(B)**: IL-3, IL-4, IL-5, IL-6, and SCF. The expression of each target gene was normalized to that of glyceraldehyde-3-phosphate dehydrogenase (GAPDH) and was expressed relative to the control sample. CSF: colony stimulating factor; IL: interleukin; SCF: stem cell factor. Data are shown as mean ± S.E.M. (n = 8); #*P* < 0.05; ** or ##*P* < 0.01 [* vs. Cont; # vs. Oxa (Steel’s multiple comparison test)].

**FIGURE 3:**
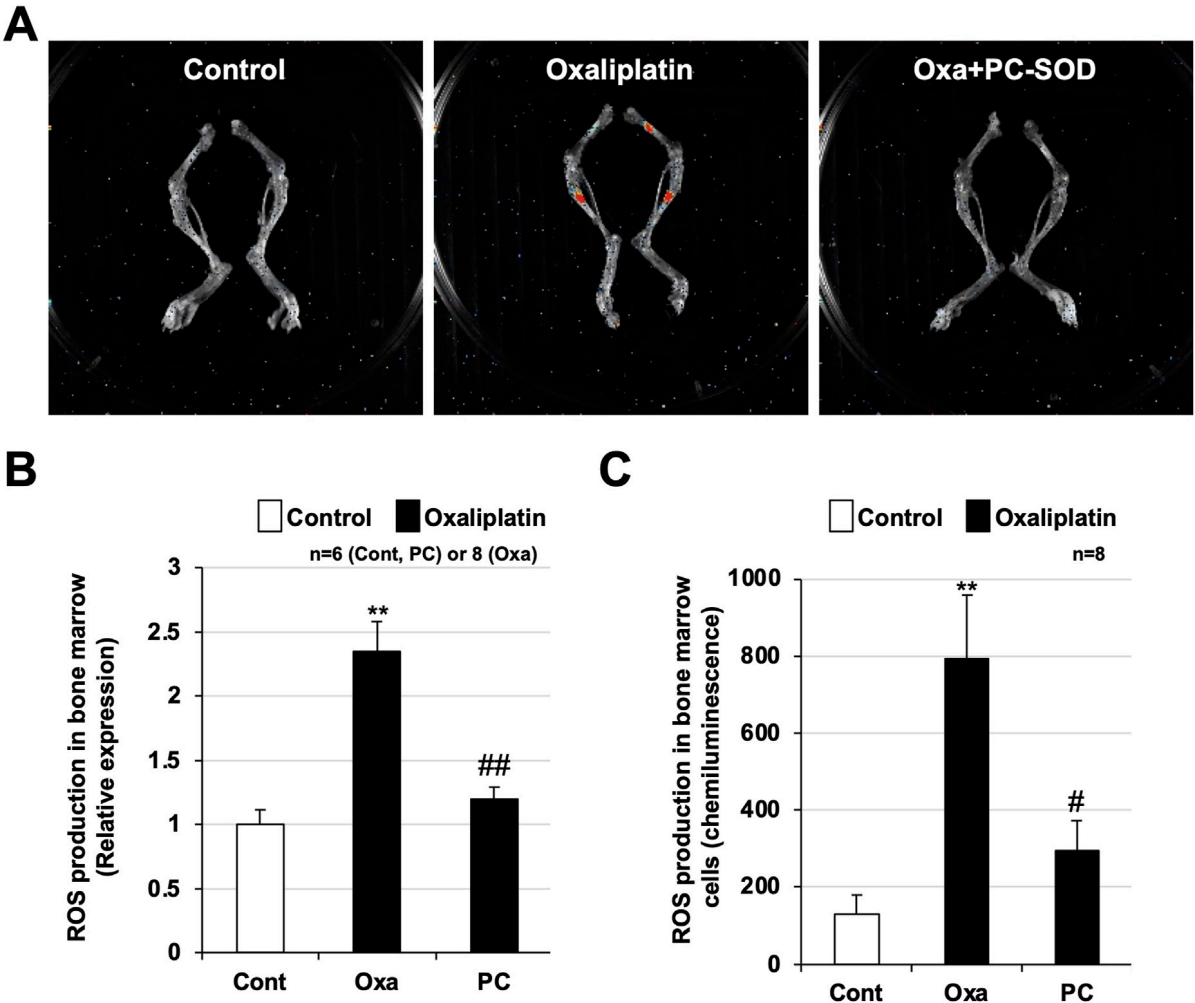
Preventive effect of PC-SOD pre-treatment on oxaliplatin-dependent reactive oxygen species (ROS) production in bone marrow. PC-SOD (PC, 3 kU/kg) or 0.45% NaCl was administered intravenously to ICR mice once daily, for a total of one time during day 1 **(A,B)** or four times during days 0–3. Oxaliplatin (Oxa, 12.5 mg/kg) or sterile saline (Cont) was administered intraperitoneally to ICR mice once on day 1. **(A)** 24 h after oxaliplatin administration, a chemiluminescent reagent (L-012, 75 mg/kg dissolved in sterile saline) was subcutaneously administered to the mice. After 20 min, the right femur of each mouse was removed. The femur was placed in a plastic Petri dish, and bright-field and chemiluminescence images (5-min exposure) were obtained using a Fusion chemiluminescence imaging system. **(B)** The chemiluminescence intensity of each sample was calculated using the software provided with the system. **(C)** 72 h after oxaliplatin administration, the right femur was removed from each mouse, and bone marrow cells were collected. The cells were homogenized by ultrasonication and hydrogen peroxide in the supernatant was measured using a hydrogen peroxide chemiluminescent detection kit. Data are shown as mean ± S.E.M. (B: n = 6-8, C: n = 8); #*P* < 0.05; ** or ##*P* < 0.01 [* vs. Cont; # vs. Oxa (Steel’s multiple comparison test)].

**FIGURE 4:**
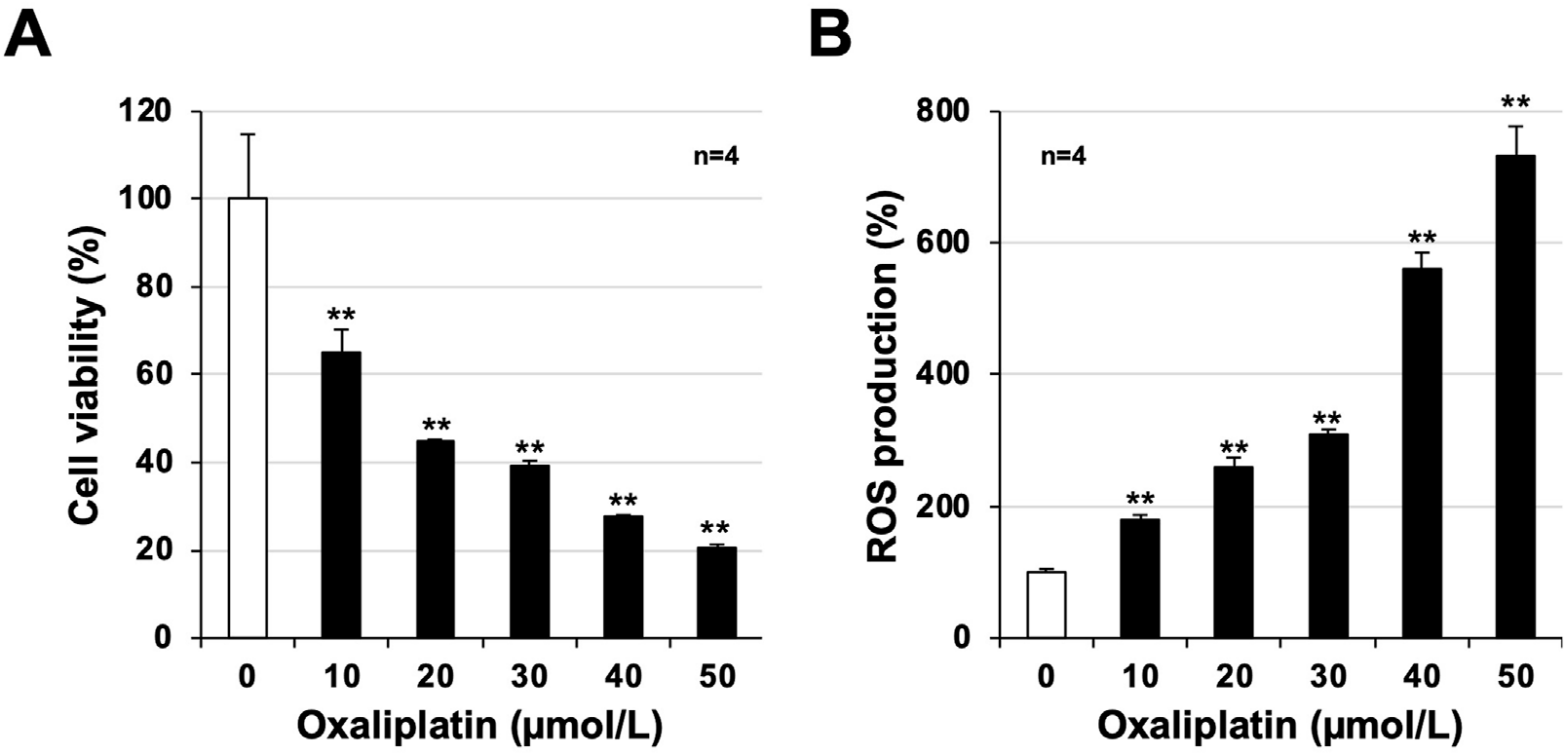
Toxicity and reactive oxygen species (ROS) production after oxaliplatin treatment in mouse-derived bone marrow cells. Bone marrow cells were harvested from untreated healthy mice and cultured in 96-well plates. After 24 h, the cells were treated with oxaliplatin (µmol/L) at the indicated concentrations and cultured for another 24 h. **(A)** Viability of bone marrow cells was determined using CellTiter-Glo^®^ 2.0 assay. **(B)** After 24 h, the cells were treated with oxaliplatin at the indicated concentrations (µmol/L) and cultured for 24 h. Oxaliplatin-treated bone marrow cells were treated with 2′,7′-dichlorodihydrofluorescein diacetate (H_2_DCFDA, 10 μmol/L) for 60 min, and the fluorescence emitted based on intracellular ROS production was measured using a microplate reader. Data are shown as mean ± S.E.M. (n = 4); #*P* < 0.05; ** or ##*P* < 0.01 [* vs. Cont; # vs. Oxa (Dunnett’s test)].

**FIGURE 5:**
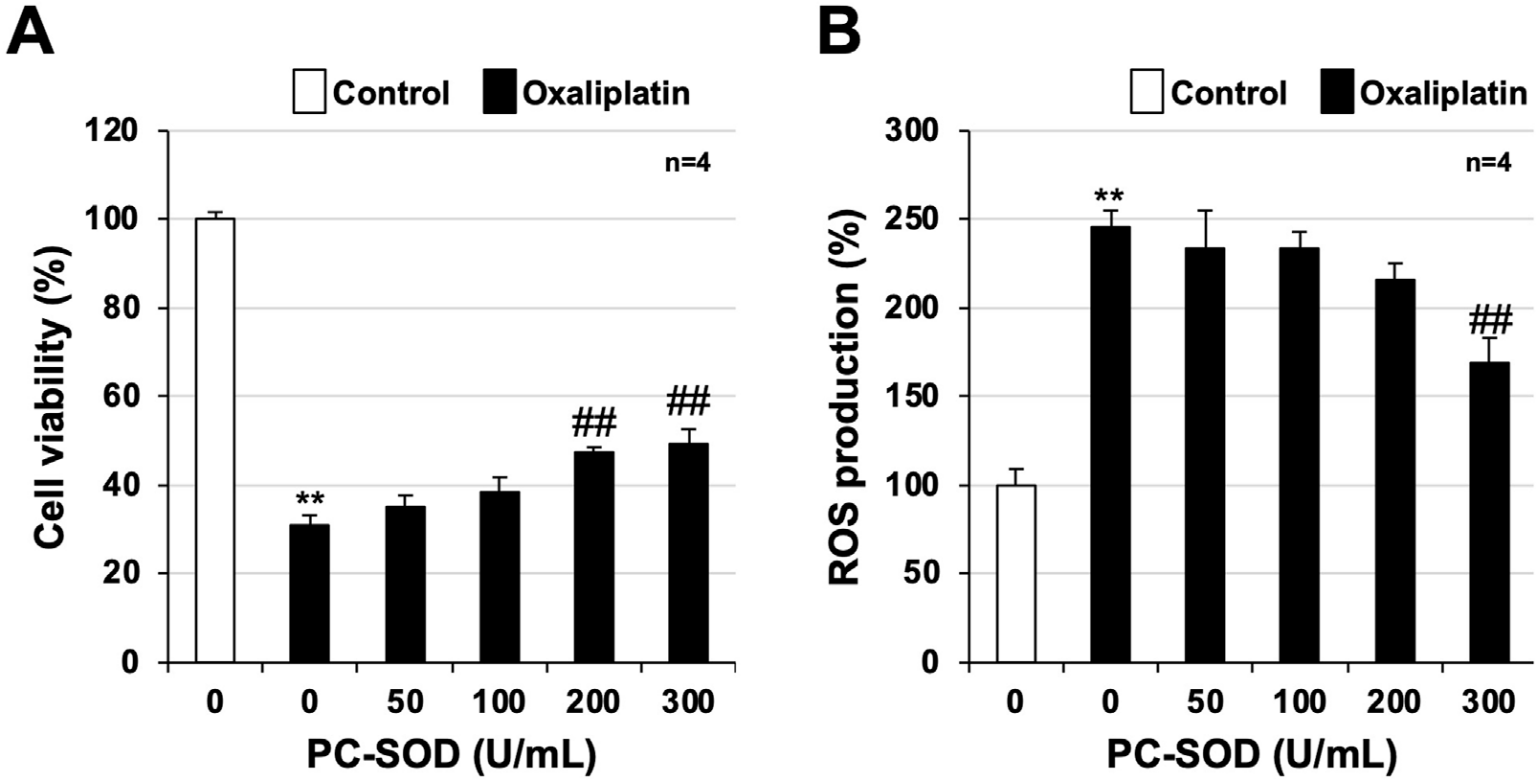
Efficacy of PC-SOD pre-treatment in mouse-derived bone marrow cells. Bone marrow cells were harvested from untreated healthy mice and cultured in 96-well plates. After 24 h, the cells were first treated with PC-SOD (U/mL) and then oxaliplatin (20 μmol/L) at the indicated concentrations and incubated for another 24 h. **(A)** Viability of bone marrow cells was determined using CellTiter-Glo^®^ 2.0. **(B)** After 24 h, cells were treated with oxaliplatin at the indicated concentrations (µmol/L) and cultured for 24 h. Oxaliplatin-treated bone marrow cells were treated with 2′,7′-dichlorodihydrofluorescein diacetate (H_2_DCFDA, 10 μmol/L) for 60 min, and the fluorescence emitted based on intracellular reactive oxygen species (ROS) production was measured using a microplate reader. Data are shown as mean ± S.E.M. (n = 4); #*P* < 0.05; ** or ##*P* < 0.01 [* vs. Cont; # vs. Oxa, (Dunnett’s test)].

All values are expressed as mean ± S.E.M. The Mann–Whitney U test or Kruskal–Wallis test, followed by Steel’s multiple comparison test, was used to evaluate differences between groups in animal experiments (Figures 1–3). One-way ANOVA followed by Dunnett’s test was used to evaluate the differences between groups in the cellular experiments (Figures 4, 5). Mac Statistical Analysis Ver. 3.0 (ESUMI Co., Ltd., Tokyo, Japan) was used for all the statistical analyses. *P* < 0.05 indicated statistical significance.

## Results

### Oxaliplatin-induced leukopenia and the effect of PC-SOD pre-administration

Oxaliplatin was administered once on day 1, and PC-SOD was administered once daily from day 0 to day 4, for a total of five doses. WBC and PLT counts decreased on days 4, 7, and 10 after oxaliplatin treatment, with a slight recovery observed on day 10 (Figure 1). RBC count and HGB levels decreased on days 4, 7, and 10 after oxaliplatin treatment. These two indices showed no recovery trend on day 10. In contrast, a trend toward improvement in oxaliplatin-dependent WBC count reduction was observed on days 4 and 7 with prophylactic administration of PC-SOD, and the reduction was significantly improved on day 10 (*P* = 0.007). PC-SOD administration significantly improved the oxaliplatin-dependent decrease in PLT count on day 7 (*P* = 0.006), although the effect was not as pronounced as the recovery in the WBC count. PC-SOD had little effect on the oxaliplatin-dependent reductions in RBC counts and HGB levels. These results suggest that PC-SOD exerts a prophylactic effect against oxaliplatin-induced myelosuppression, especially leukopenia.

### Decreased expression of cytokines involved in hematopoietic progenitor cell differentiation and proliferation and the effect of PC-SOD pre-administration

Next, we analyzed the gene expression of colony-stimulating factor 2 and 3 [CSF-2 and CSF3, also known as granulocyte-macrophage (GM)-CSF and G-CSF], glycoprotein hormones required for hematopoietic progenitor cell differentiation and proliferation (Ogawa, 1993; Huang et al., 1999), using real-time RT-PCR. Bone marrow cells were harvested from the bones of both legs of mice on day 10, RNA was purified, and cDNA was synthesized and used for analyses. Significant downregulation of CSF-2 and CSF-3 mRNAs was observed in bone marrow cells from oxaliplatin-treated mice compared with bone marrow cells from control mice (Figure 2A). This downregulation was significantly ameliorated in the bone marrow cells from mice pre-treated with PC-SOD (CSF-2: *P* = 0.001, CSF-3: *P* = 0.001). We also examined the gene expression of other cytokines associated with hematopoietic progenitor cell differentiation and proliferation (Ogawa, 1993; Huang et al., 1999) and found that the gene expression of interleukin (IL)-3, IL-4, IL-5, IL-6, IL-9, and stem cell factor (SCF) was significantly downregulated by oxaliplatin treatment compared with that in the controls, whereas pre-treatment with PC-SOD significantly reversed this downregulation (IL-3: *P* = 0.004, IL-4: *P* = 0.020, IL-5: *P* = 0.003, IL-6: *P* = 0.020, IL-9: *P* = 0.002, SCF: *P* = 0.006) (Figure 2B). These results indicate that PC-SOD exerts a prophylactic effect against oxaliplatin-induced leukopenia.

### Oxaliplatin-induced ROS production and the effect of PC-SOD pre-administration

Next, we used an *in vivo* imaging system to analyze ROS production in the leg bones of mice. A chemiluminescent reagent that reacts with ROS was subcutaneously administered to the mice 24 h after oxaliplatin administration, and both leg bones were removed 20 min later. In mice administered oxaliplatin, red-stained areas were detected in the central part of the femur and tibia, indicating areas of increased ROS production (Figure 3A). Pre-administration of PC-SOD markedly suppressed oxaliplatin-induced ROS production in the central part of the femur and tibia. ROS production was increased 2.5 ± 0.3-fold by oxaliplatin and reduced to 1.2 ± 0.3-fold by PC-SOD pre-treatment (*P* = 0.002) (Figure 3B). We further analyzed ROS production in bone marrow cells from mice in the control, oxaliplatin-treated, and PC-SOD + oxaliplatin groups. As shown in Figure 3C, bone marrow cells from the oxaliplatin-treated group showed marked ROS production, whereas bone marrow cells from mice pre-treated with PC-SOD followed by oxaliplatin treatment showed significantly reduced ROS production (*P* = 0.025). These results suggest that PC-SOD suppresses oxaliplatin-induced leukopenia by inhibiting ROS production in bone marrow cells.

### Analysis of toxicity and ROS production in normal bone marrow cells in mice

Finally, we analyzed oxaliplatin-dependent toxicity and ROS production in an experimental cellular system using bone marrow cells from untreated healthy mice. We also analyzed whether PC-SOD directly inhibits oxaliplatin-dependent toxicity and ROS production *in vitro*. Bone marrow cells cultured for 24 h were treated with oxaliplatin (final concentration: 10–60 μmol/L), and cytotoxicity and ROS production were measured after another 24 h (Figures 4A,B). Oxaliplatin showed toxicity to bone marrow cells, and cell viability decreased in a concentration-dependent manner with oxaliplatin (Figure 4A). We also measured intracellular ROS production using H_2_DCFDA and found a significant increase in intracellular ROS production that was dependent on the oxaliplatin concentration (Figure 4B).

Next, we examined the effect of PC-SOD pre-treatment on cytotoxicity and ROS production with 20 μmol/L of oxaliplatin. The oxaliplatin-induced reduction in cell viability was restored by PC-SOD pre-treatment, with significant recovery at 200 U/mL and 300 U/mL PC-SOD pre-treatment (200 U/mL: *P* = 0.001, 300 U/mL: *P* < 0.001) (Figure 5A). Oxaliplatin-induced intracellular ROS production was inhibited in a concentration-dependent manner by PC-SOD pre-treatment, with 300 U/mL PC-SOD pre-treatment showing a significant inhibitory effect (*P* = 0.001) (Figure 5B). These results suggest that PC-SOD exerts a direct inhibitory effect on oxaliplatin-induced cytotoxicity and ROS production in bone marrow cells.

## Discussion

PC-SOD has been shown to be safe and well tolerated in healthy subjects and effective for several diseases (Kamio et al., 2014; Chen et al., 2019). In this study, we examined the potential preventive effects of intravenous PC-SOD in a mouse model of oxaliplatin-induced leukopenia. We found that prophylactic PC-SOD administration markedly suppressed leukopenia by inhibiting oxaliplatin-dependent ROS production in the mouse bone marrow. In our study on PC-SOD for oxaliplatin-induced peripheral neuropathy, we showed that PC-SOD did not affect the anticancer activity of oxaliplatin. Subcutaneous transplantation of HCT116 human colon adenocarcinoma cells into mice resulted in increased tumor volume, which was suppressed equally in the FOLFOX + vehicle and FOLFOX + PC-SOD groups, suggesting PC-SOD had no effect on oxaliplatin’s anticancer activity (Qiao et al., 2024). Furthermore, a recent phase II clinical trial demonstrated PC-SOD’s efficacy in oxaliplatin-dependent chemotherapy-induced peripheral neuropathy across several endpoints (https://www.ltt.co.jp/en/, accessed 20 December 2024). These results suggest that PC-SOD prevents oxaliplatin-dependent myelosuppression, particularly leukopenia, without affecting the anticancer activity of oxaliplatin.

Analysis using an *in vivo* imaging system showed that intraperitoneal oxaliplatin enhanced ROS production in the central femur and tibia of mice (bone-marrow). Prophylactic administration of PC-SOD was effective against ROS production in mouse bone marrow. The observation of increased ROS production in the bone marrow in an oxaliplatin-dependent myelosuppression model and the finding of prophylactic efficacy are novel findings deserving emphasis. We confirmed an oxaliplatin-dependent increase in ROS production in mouse bone marrow cells and found that PC-SOD directly suppressed this production. Another study reported that calmangafodipir, a mimetic of manganese SOD, prevented oxaliplatin-dependent myelosuppression; however, the authors did not analyze oxidative stress, including ROS production (Karlsson et al., 2012). This may be due to the difficulty in detecting ROS production in the oxaliplatin-dependent myelosuppression model. Although the detection of ROS was difficult, we succeeded by adjusting the experimental conditions based on our previous studies (Tanaka et al., 2017; Tanaka et al., 2021; Tanaka et al., 2022a; Tanaka et al., 2022b). These results are important for the clinical application of PC-SOD as a prophylactic agent.

G-CSF is used to treat neutropenia and febrile neutropenia caused by cancer chemotherapy by promoting granulocyte differentiation and proliferation (Cooper et al., 2011). Clinical trials have shown that polyethylene glycol-conjugated G-CSF prevents neutropenia and febrile neutropenia in patients receiving oxaliplatin-containing chemotherapy (Kitagawa et al., 2020; Macaire et al., 2020). While PC-SOD suppressed CSF2 and CSF3 expression in mouse bone marrow cells, we believe that this effect was indirect, as it prevented oxaliplatin-induced cell injury. *In vitro* experiments showed that prophylactic PC-SOD administration inhibited oxaliplatin-induced cell death and ROS production in bone marrow cells. Given these results, we speculate that PC-SOD’s mechanism of action of PC-SOD differs from that of G-CSF used in clinical practice. Thus, PC-SOD and G-CSF can be combined to prevent anticancer drug-induced myelosuppression. Future studies should analyze their combined effects in animal models.

Oxaliplatin is used clinically as a single agent and in the FOLFOX protocol with 5-fluorouracil and leucovorin and the XELOX protocol with capecitabine (Comella et al., 2009; Rottenberg et al., 2021). Cisplatin and gemcitabine are also used clinically. Myelosuppression is a serious adverse effect of these combinations and other anticancer drugs (Bjorn et al., 2022; Hart et al., 2023). In animal models, ω-3 polyunsaturated fatty acids inhibit cisplatin-induced myelosuppression via Nrf2-mediated antioxidant induction (Xu et al., 2023). Quercetin inhibits cisplatin-induced myelosuppression, whereas carnosine inhibits cyclophosphamide-induced bone marrow suppression (Xu et al., 2014; Chuang et al., 2022). These findings suggest that antioxidant compounds may prevent myelosuppression induced by various anticancer agents. Therefore, PC-SOD may prevent myelosuppression caused by oxaliplatin and other anticancer agents.

## Conclusion

The current study is the first to demonstrate that PC-SOD significantly suppresses oxaliplatin-dependent myelosuppression, particularly leukopenia, in a mouse model by exerting an antioxidant effect. Although several issues remain to be addressed, these results may lead to the clinical application of PC-SOD as a method for preventing myelosuppression caused by oxaliplatin administration.

## Data Availability

The original contributions presented in the study are included in the article/Supplementary Material, further inquiries can be directed to the corresponding author.

## Abbreviations

CSF, colony stimulating factor; GAPDH, glyceraldehyde-3-phosphate dehydrogenase; G-CSF, granulocyte colony stimulating factor; H2DCFDA, dichlorodihydrofluorescein diacetate; HGB, hemoglobin; IL, interleukin; MDA, malondialdehyde; PC-SOD, lecithinized superoxide dismutase; PLT, platelet; ROS, reactive oxygen species; RBC, red blood cell; SCF, stem cell factor; WBC, white blood cell.
