# Supplementary material for: Prophylactic administration of lecithinized superoxide dismutase for a murine model of oxaliplatin-induced myelosuppression
Source: Front Pharmacol. 2025 Jul 22;16:1607814. doi: 10.3389/fphar.2025.1607814 (PMC12322972; doi:10.3389/fphar.2025.1607814)
Supplement: Supplementary file 1 [file DataSheet1.pdf]

Gene	Primer	Sequence (5' to 3')
**CSF2**	Forward	AACCTCCTGGATGACATGCCTG
	Reverse	AAATTGCCCCGTAGACCCTGCT
**CSF3**	Forward	ATCCCGAAGGCTTCCCTGAGTG
	Reverse	AGGAGACCTTGGTAGAGGCAGA
**IL-3**	Forward	CCTGCCTACATCTGCGAATGAC
	Reverse	GAGGTTAGCACTGTCTCCAGATC
**IL-4**	Forward	CCAAGGTGCTTCGCATATTT
	Reverse	ATCGAAAAGCCCGAAAGAGT
**IL-5**	Forward	GTGGGGGTACTGTGGAAATG
	Reverse	TAATCCAGGAACTGCCTCGT
**IL-6**	Forward	CTGGAGTCACAGAAGGAGTGG
	Reverse	GGTTTGCCGAGTAGATCTCAA
**IL-9**	Forward	ACCACATGGGGCATCAGAGA
	Reverse	TCATCAGTTGGGACGGAGAG
**SCF**	Forward	ATCTGCGGGAATCCTGTGAC
	Reverse	CATCCCGGCGACATAGTTGA
**GAPDH**	Forward	AACTTTGGCATTGTGGAAGG
	Reverse	ACACATTGGGGGTAGGAACA

Supplementary Figure S1. List of primer sequences used
